# Intrafraction stability using full head mask for brain stereotactic radiotherapy

**DOI:** 10.1002/acm2.13382

**Published:** 2021-08-04

**Authors:** Jun Tomihara, Jun Takatsu, Satoru Sugimoto, Naoto Shikama, Keisuke Sasai

**Affiliations:** ^1^ Department of Radiation Oncology, Graduate School of Medicine Juntendo University Tokyo Japan; ^2^ Department of Radiology Juntendo University Hospital Tokyo Japan; ^3^ Department of Radiation Oncology Juntendo University Tokyo Japan

**Keywords:** interfractional setup error, intrafractional setup error, mouthpiece, planning target volume margin, stereotactic radiosurgery

## Abstract

**Purpose:**

We investigated the immobilization accuracy of a new type of thermoplastic mask—the Double Shell Positioning System (DSPS)—in terms of geometry and dose delivery.

**Methods:**

Thirty‐one consecutive patients with 1–5 brain metastases treated with stereotactic radiotherapy (SRT) were selected and divided into two groups. Patients were divided into two groups. One group of patients was immobilized by the DSPS (n = 9). Another group of patients was immobilized by a combination of the DSPS and a mouthpiece (n = 22). Patient repositioning was performed with cone beam computed tomography (CBCT) and six‐degree of freedom couch. Additionally, CBCT images were acquired before and after treatment. Registration errors were analyzed with off‐line review. The inter‐ and intrafractional setup errors, and planning target volume (PTV) margin were also calculated. Delivered doses were calculated by shifting the isocenter according to inter‐ and intrafractional setup errors. Dose differences of GTV D_99%_ were compared between planned and delivered doses against the modified PTV margin of 1 mm.

**Results:**

Interfractional setup errors associated with the mouthpiece group were significantly smaller than the translation errors in another group (*p* = 0.03). Intrafractional setup errors for the two groups were almost the same in all directions. PTV margins were 0.89 mm, 0.75 mm, and 0.90 mm for the DSPS combined with the mouthpiece in lateral, vertical, and longitudinal directions, respectively. Similarly, PTV margins were 1.20 mm, 0.72 mm, and 1.37 mm for the DSPS in the lateral, vertical, and longitudinal directions, respectively. Dose differences between planned and delivered doses were small enough to be within 1% for both groups.

**Conclusions:**

The geometric and dosimetric assessments revealed that the DSPS provides sufficient immobilization accuracy. Higher accuracy can be expected when the immobilization is combined with the use of a mouthpiece.

## 1 INTRODUCTION

Linac‐based stereotactic radiosurgery (SRS) has been used extensively in modern radiotherapy for brain metastases.^1^ Volumetric modulated arc therapy (VMAT) SRS and fractionated stereotactic radiotherapy (SRT) can generate steep dose gradients and spare adjacent normal tissues.^2^, ^3^ Furthermore, the implementation of flattening filter‐free (FFF) beams was also the main factor for the utilization of linac‐based SRS for treating brain metastases.^4^ Therefore, the accuracy of target localization for delivering brain SRS and SRT has been investigated.^1^, ^2^, ^3^, ^5^, ^6^, ^7^, ^8^, ^9^, ^10^, ^11^, ^12^ Kirkpatrick et al. claimed that the 1‐mm planning target volume (PTV) margin is more appropriate to avoid radionecrosis for brain SRS.^2^ Immobilization with a head ring was used for brain SRS, but the invasiveness associated with this process was a burden on patients. In recent years, many studies have reported the setup accuracy for brain SRS/SRT in conjunction with the use of a thermoplastic mask, instead of the accuracy associated with the invasive conventional head ring procedure.^1^, ^3^, ^6^, ^7^, ^8^, ^9^, ^10^, ^11^, ^12^

Uncertainty in the reproducibility of the jaw position due to the thermoplastic mask has been reported.^11^, ^12^ Still, methods to reduce this uncertainty by combining the thermoplastic mask with a fixation device has been reported.^6^, ^7^, ^11^, ^12^ Babic et al. have investigated the improvement of inter/intrafractional setup uncertainties with a mouthpiece.^12^ In addition, they found that the immobilization accuracy decreased as the number of treatment fractions increased.^12^ Additionally, the immobilization accuracy depends on the type of frameless immobilizations.^12^ Thus, brain SRT using frameless immobilization requires a prior assessment of the reproducibility of the immobilization during the treatment course.

In linac‐based SRS/SRT, the single isocenter irradiation for multiple targets shortens the treatment time. However, even a small rotation setup error causes a large underdose with the considerable distance between the isocenter and the target.^5^ Rotational errors then affect the PTV margin in the single isocenter technique.^13^ Assessment of inter‐ and intrafractional rotational errors is also essential for the assessment of the immobilization accuracy in brain SRS/SRT. Many previous studies have proposed appropriate PTV margins from a geometric point of view for brain SRS/SRT.^13^, ^14^ However, since the targets were spherical in these studies, the PTV margin for irregularly shaped targets, especially postoperative tumors, has not been sufficiently investigated. In this study, we calculated delivered dose from the registration information on CBCT taken both before and after treatment for postoperative and preoperative tumors.

The present study aimed to investigate the inter‐ and intrafractional setup accuracy of the combination of the newly developed thermoplastic mask and the mouthpiece. In addition, we also determined the PTV margin with this frameless immobilization system from a geometric standpoint. Additionally, dosimetric errors between planned and delivered doses were evaluated against the target volume and the distance from the isocenter. The DSPS and the mouthpiece were assessed for their impact on dose delivery.

## 2 METHODS

### 2.1 Immobilization and simulations

Thirty‐one consecutive patients with 1–5 brain metastases treated in a single isocenter SRT between April 2018 and March 2020 were selected for this work. Table 1 shows the characteristics of patients. All of the patients were immobilized by the Double Shell Positioning System (DSPS) (Macromedics BV, Waddinxveen, Netherlands). The DSPS fixes the patient's cranium anteriorly and posteriorly with two thermoplastic masks.^15^ Furthermore, the custom thermoplastic mouthpiece (Precise Bite^TM^ [CIVCO Medical Solutions]) was made by a radiation oncologist for 22 patients (Table 1).

**TABLE 1:** Patient characteristics

Characteristics	Values
Patients	
Mouthpiece	22 patients 32 plans
No‐mouthpiece	9 patients 14 plans
Age (mean ± SD)	67.6 ± 9.8
Gender (Male/Female)	16/15
Prescription dose	
20 Gy in 5 fractions	3 plans (6.5%)
25 Gy in 5 fractions	5 plans (10.9%)
30 Gy in 3 fractions	31 plans (67.4%)
30 Gy in 5 fractions	7 plans (15.2%)

Abbreviation: SD, standard deviation.

Planning computed tomography (CT) imaging was performed with Aquilion LB (Canon Medical Systems). The resolution of CT images was 1.07 × 1.07 mm^2^ and the slice thickness was 1.0 mm. The field of view (FOV) was 550 mm in diameter and the X‐ray tube voltage was 120 kV. Eclipse ver. 13.6 (Varian Medical Systems) was used for target delineation and RayStation ver. 6.2 (RaySearch Laboratories) was used for dose calculation with the collapsed cone algorithm. A calculation grid of 1 mm was used.

This study was approved by the ethics committee of our institution and conducted according to the Declaration of Helsinki. Informed consents from patients were obtained by “opt‐out” method.

### 2.2 Treatment planning and delivery

Gross tumor volume was contoured using longitudinal relaxation T1‐weighted magnetic resonance images (1 mm thickness). A total of 1–5 targets were treated in single isocenter SRT. The PTV margin used in this study was 2 mm or 3 mm. Prescription doses were delivered with various protocols according to the dose constraints of the normal brain^3^ (30 Gy in 3 fractions, 30 Gy in 5 fractions, 25 Gy in 5 fractions, and 20 Gy in 5 fractions). The prescription dose was normalized to 95% of PTV. All VMAT plans were generated using 6 MV flattening filter‐free (FFF) photon beams of TrueBeam (Varian Medical Systems) with a Millennium 120 multileaf collimator (MLC). The maximum dose rate was 1400 monitor units per minute. VMAT plans consisted of one full coplanar arc and two or three partial noncoplanar arcs.

Following the initial cone beam CT (initial‐CBCT) scan, repositioning was performed with the six‐degree freedom (6‐DOF) Perfect Pitch couch (Varian Medical Systems). CBCT was scanned again after position correction for the identification of the initial patient position (pre‐CBCT). Radiation therapists entered the treatment room to perform manual rotations of the couch for noncoplanar arcs. After completing the treatment, the couch was manually rotated to its initial position and CBCT was performed (post‐CBCT). CBCT images were acquired with the Varian On‐Board Imager (Varian Medical Systems). In our institution, Head (100 kV, 150 mAs) and Spotlight (125 kV, 150–650 mAs) protocols were used for CBCT imaging. The resolution of CBCT scanning was 0.51 × 0.51 mm^2^ and the slice thickness was 2.0 mm. The FOV of Head and Spotlight protocols was 261.7 mm.

### 2.3 Data analysis

The shifted bony anatomy following a rigid registration between the pre‐CBCT and the planning CT indicates the existence of an interfractional setup error. Given that the repositioning was already performed for the initial‐CBCT and 6‐DOF Perfect Pitch couch, the interfractional setup error was precisely the residual setup error. Similarly, the intrafractional setup error was obtained from the displacements between the pre‐CBCT and the post‐CBCT. Rigid registration was performed with off–ine review tool of ARIA Oncology Information system (Varian Medical Systems). Treatment time was defined as a total time spanned from the pre‐CBCT to the post‐CBCT.

To compare the immobilization accuracy of this study with other studies,^7^, ^11^ the PTV margin was calculated based on the study by van Herk et al.^16^ The recipe regulated the margin size that assumes a complete conformal dose distribution for clinical target volume (CTV) based on the “rolling ball algorithm.” The PTV margin recipe was as follows:

(1) $${\text{van Herk}}^{\prime }\;\text{s recipe}:\;\text{PTV margin}\;=\;2\text{.5}\Sigma +0\text{.7}\sigma$$

The first term “Σ” is a vector comprising the standard deviations of all systematic error sources. The second term “σ” is a vector comprising the standard deviations of all sources for random errors. In the van Herk's recipe, it uses numbers that aim to deliver a dose of at least 95% of CTV in 90% of the patients.

Given that the PTV margin calculation needs to consider both inter‐ and intrafractional setup errors, the following relational expression was added. To assume the worst case of setup errors, the PTV margin for considering both interfractional and intrafractional setup errors was defined as follows:

(2) PTV margin(Sum)=PTV margin(Inter)+PTV margin(Intra)

### 2.4 Statistical analyses

Two tailed *t*‐tests were used to analyze the statistical difference of inter‐ and intrafractional setup errors between the two groups (with and without the mouthpiece). Statistical significance of treatment time between the two groups was also analyzed. All *p* values were considered significant at *p* < 0.05. Statistical analyses were performed with the software R ver. 3.6.1 (R Foundation).

### 2.5 Delivered dose analyses

Delivered doses were reconstructed using pre‐ and post‐CBCT rigid registration data. Similarly, to calculate the PTV margin, we assumed the worst case and calculated delivered doses that included inter‐ and intrafractional setup errors. To evaluate the immobilization accuracy of the DSPS and the mouthpiece, we created modified treatment plans with a PTV margin of 1 mm (modified PTV). The beam arrangements, beam energy, and calculation grid were the same as those used in clinical practice. The prescription dose of the original clinical plan was normalized to 95% of the modified PTV. Gradient index^17^ (GI) and Paddick's conformity index^18^ (PCI) were used to quantitatively evaluate the quality of planned dose distributions.

Delivered doses were calculated by shifting the isocenter position at each fraction on the planning CT with RayStation software. We then assessed the differences in GTV D_99%_ and PTV D_95%_ between the combined delivered doses and planned doses. To assess dosimetric errors when SRS was performed using the DSPS and mouthpiece, comparisons of GTV D_99%_ and PTV D_95%_ were performed between planned doses and delivered doses on the first fraction.

## 3 RESULTS

**FIGURE 1:**
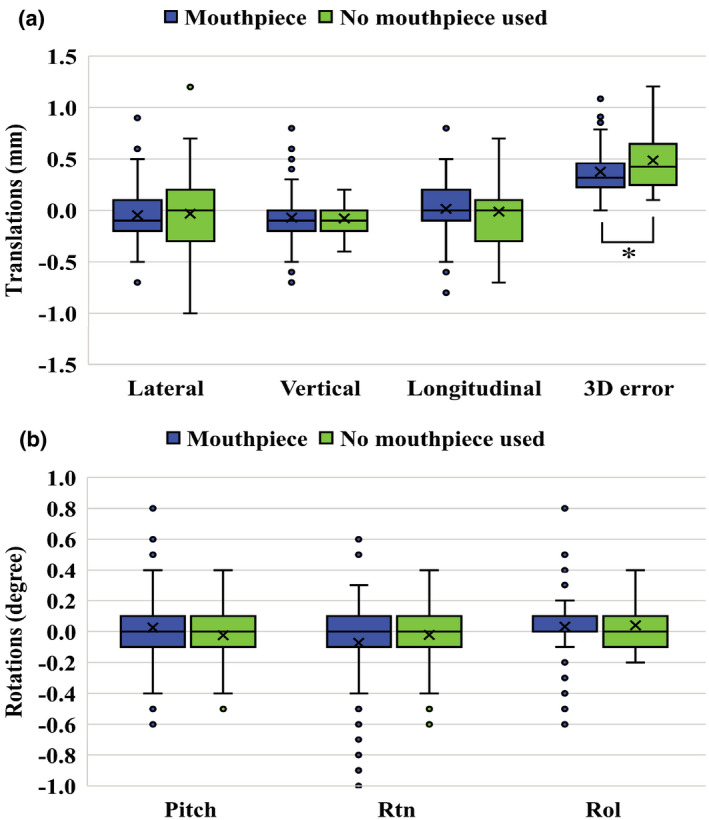
Box plots of interfractional setup errors for translations (a) and rotations (b). Box plots represent upper and lower quartiles, and the ends of the whiskers represent 1.5× interquartile or maximum ranges. Circles represent outliers, and central solid lines and crosses represent the median and mean values, respectively. The three‐dimensional error is the root square sum value of the errors in the lateral, vertical, and longitudinal directions. Significant differences (*p* < 0.05) between the two groups are marked by an asterisk

A total of 31 patients with 151 image sets were analyzed. Table 2 summarizes the interfractional setup errors in each translation and rotation (translations include lateral, vertical, and longitudinal directions; rotations include Pitch, Rtn, and Rol directions). The three‐dimensional interfractional setup error of the no‐mouthpiece group was significantly larger than that of another group regarding the three‐dimensional (3D) error (*p* = 0.03). Figure 1 shows the box plots of interfractional setup errors for translations and rotations.

**TABLE 2:** Average, standard deviation, and maximum interfractional setup errors in each direction and angle

	Translations (mm)				Rotations (°)		
	Lateral	Vertical	Longitudinal	3D error	Pitch	Rtn	Rol
Mouthpiece (N = 109)							
Average	0.0	−0.1	0.0	0.4	0.0	−0.1	0.0
SD	0.2	0.2	0.3	0.2	0.2	0.3	0.2
Maximum	0.9	0.8	0.8	1.1	0.8	1.0	0.8
No‐mouthpiece (N = 37)							
Average	0.0	−0.1	0.0	0.5	0.0	0.0	0.0
SD	0.4	0.2	0.4	0.3	0.2	0.2	0.1
Maximum	1.2	0.4	0.7	1.2	0.5	0.6	0.4
*p* value	0.82	0.85	0.67	**0.03**	0.21	0.29	0.76

Abbreviations: Maximum = Absolute maximum error; SD = Standard deviation; Three‐dimensional error = Root square sum of lateral, vertical, and longitudinal values. Bold type means significant difference.

The averages of interfractional setup errors were almost the same between the two groups regarding translations and rotations, respectively. The standard deviations of the interfractional setup errors were decreased when the mouthpiece was used in the lateral and longitudinal directions.

**FIGURE 2:**
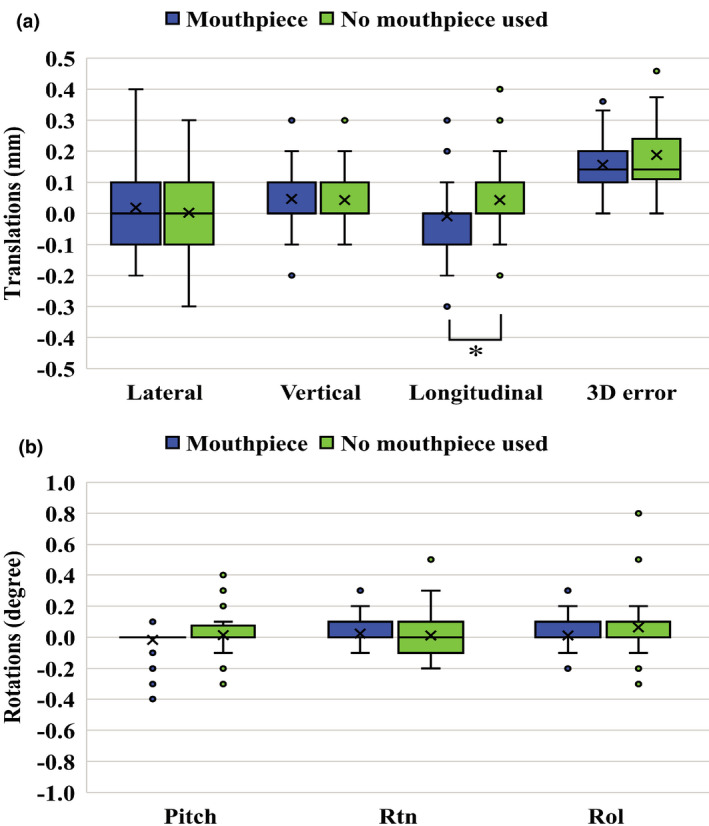
Box plots of intrafractional setup errors for translations (a) and rotations (b). Box plots represent upper and lower quartiles, and the ends of the whiskers represent 1.5× interquartile or maximum ranges. Circles represent outliers, and central solid lines and crosses represent median and mean values, respectively. The three‐dimensional error is the root square sum value of the errors in the lateral, vertical, and longitudinal directions. Significant differences (*p* < 0.05) between the two groups are marked by an asterisk

Table 3 summarizes the intrafractional setup errors in each translation and rotation. The intrafractional longitudinal error of the mouthpiece group was significantly smaller than that of another group (*p* = 0.02). Figure 2 shows the box plots of intrafractional setup errors for translations and rotations.

**TABLE 3:** Average, standard deviation, and maximum intrafractional setup errors in each direction and angle

	Translations (mm)				Rotations (degree)		
	Lateral	Vertical	Longitudinal	3D error	Pitch	Rtn	Rol
Mouthpiece (N = 101)							
Average	0.0	0.0	0.0	0.2	0.0	0.0	0.0
SD	0.1	0.1	0.1	0.1	0.1	0.1	0.1
Maximum	0.4	0.3	0.3	0.6	0.4	0.3	0.3
No‐mouthpiece (N = 42)							
Average	0.0	0.0	0.0	0.2	0.0	0.0	0.1
SD	0.1	0.1	0.1	0.1	0.1	0.1	0.2
Maximum	0.3	0.3	0.4	0.5	0.4	0.5	0.8
*p* value	0.49	0.81	**0.02**	0.21	0.17	0.60	0.73

Abbreviations: Maximum = Absolute maximum error; SD = Standard deviation; Three‐dimension error = Root square sum of lateral, vertical, and longitudinal values. Bold type means significant difference.

The use of the mouthpiece improved the absolute maximum error of the intrafractional setup errors in Rtn and Rol. Even though the *p* value of intrafractional longitudinal error was 0.02, the difference between the two groups was less or equal to 0.1 mm. The mean and the standard deviation of the intrafractional setup errors were also less or equal to 0.2 mm. Therefore, the difference between the two groups was almost the same in all directions. High immobilization accuracy can be achieved irrespective of the use of the mouthpiece in intrafractional setup motions.

The mean treatment times were 488 ± 69 s and 495 ± 114 s for the mouthpiece and no‐mouthpiece groups, respectively. No significant difference existed between the two groups regarding treatment time (*p* = 0.71).

Table 4 demonstrates the reported intrafractional setup errors without invasive immobilization devices. Comparing with previous studies,^6^, ^12^, ^19^ a head and shoulder thermoplastic mask is more accurate than the head thermoplastic mask alone. The use of a head thermoplastic mask in combination with the mouthpiece also reduces errors. Intrafractional setup errors of the DSPS were smaller than the Brainlab thermoplastic mask that also fix a patient's posterior head similar as the DSPS.^19^

**TABLE 4:** Summary of intrafractional setup errors without invasive immobilization devices

References	Immobilization	3D error ± SD (mm)	Number of patients (Number of scans)
Tryggestad et al.^6^	Thermoplastic (head)	1.1 ± 1.2	20 (462)
	Thermoplastic (head) + body cast	1.1 ± 1.1	9 (218)
	Thermoplastic (head and shoulder)	0.7 ± 0.9	81 (1743)
	Thermoplastic (head and shoulder) + mouthpiece	0.7 ± 0.8	11 (254)
Babic et al.^12^	Thermoplastic (head)	0.8 ± 0.5	32 (1491)
	Thermoplastic (head) + mouthpiece	0.3 ± 0.2	15 (45)
Lesiuk et al.^19^	Brainlab thermoplastic mask	0.66	12 (303)
Present study	DSPS	0.2 ± 0.1	9 (37)
	DSPS +mouthpiece	0.2 ± 0.1	22 (109)

Abbreviations: Thermoplastic = Thermoplastic mask; DSPS = Double Shell Positioning System; SD = Standard deviation; Three‐dimension error = Root square sum of lateral, vertical, and longitudinal values

Table 5 summarizes the PTV margins according to van Herk's recipe in each direction. The PTV margins were smaller than 1.0 mm in the mouthpiece group case. Conversely, the PTV margins of the no‐mouthpiece group were less than 1.5 mm. In all directions, our immobilization systems achieved high precise immobilization, especially when the mouthpiece was combined with the DSPS.

**TABLE 5:** Inter‐ and Intra‐systematic errors and random errors for PTV margin calculations

	Mouthpiece (mm)			No‐mouthpiece (mm)		
	Lateral	Vertical	Longitudinal	Lateral	Vertical	Longitudinal	
Σ (Inter)	0.2	0.2	0.2	0.3	0.1	0.3	
Σ (Intra)	0.1	0.1	0.1	0.1	0.1	0.1	
σ (Inter)	0.2	0.2	0.2	0.3	0.1	0.3	
σ (Intra)	0.1	0.1	0.1	0.1	0.1	0.1	
PTV margin (Sum)	0.89	0.75	0.90	1.20	0.72	1.37	

Abbreviations: Σ = Systematic error; σ = Random error.

**FIGURE 3:**
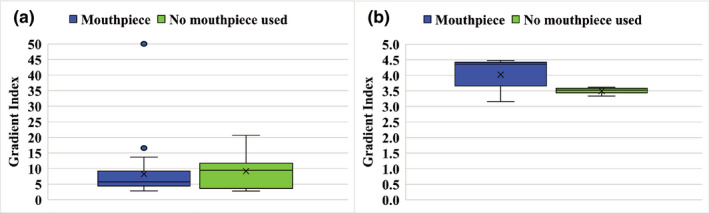
Box plots of Gradient index for all plans (a) and target volume more than 3 cc (b). Box plots represent upper and lower quartiles, and the ends of the whiskers represent 1.5× interquartile or maximum ranges. Circles represent outliers, and central solid lines and crosses represent the median and mean values, respectively

Figure 3 shows GI values of dose distribution against modified PTVs. The GI values were 8.25 ± 8.56 and 9.15 ± 5.88 for the mouthpiece and no‐mouthpiece groups, respectively. When limited to cases with the PTV volume of 3 cc or more, the GI values were 4.02 ± 0.53 and 3.50 ± 0.13 for the mouthpiece and no‐mouthpiece groups, respectively. The CI values were 0.84 ± 0.11 and 0.81 ± 0.06 for the mouthpiece and no‐mouthpiece groups, respectively.

**FIGURE 4:**
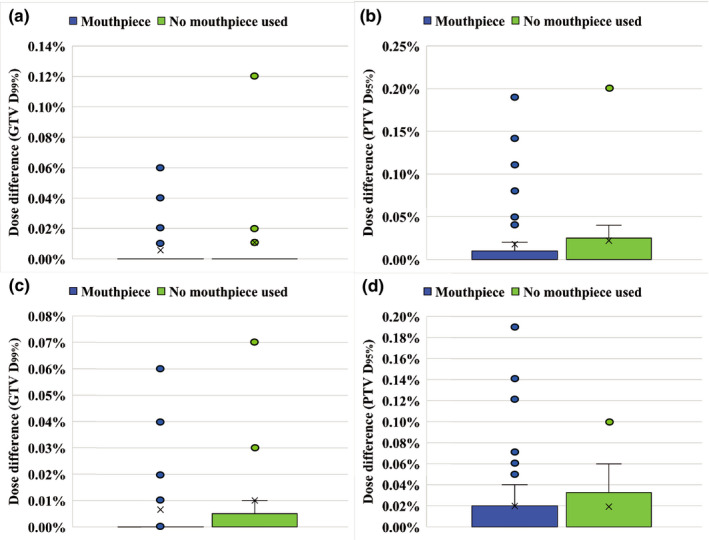
Dose differences between planned and delivered doses for two groups. GTV D_99%_ (a) and PTV D_95%_ (b) for SRT technique; GTV D_99%_ (c) and PTV D_95%_ (d) for SRS technique. Box plots represent upper and lower quartiles, and the ends of the whiskers represent 1.5× interquartile or maximum ranges. Circles represent outliers, and central solid lines and crosses represent the median and mean values, respectively

**FIGURE 5:**
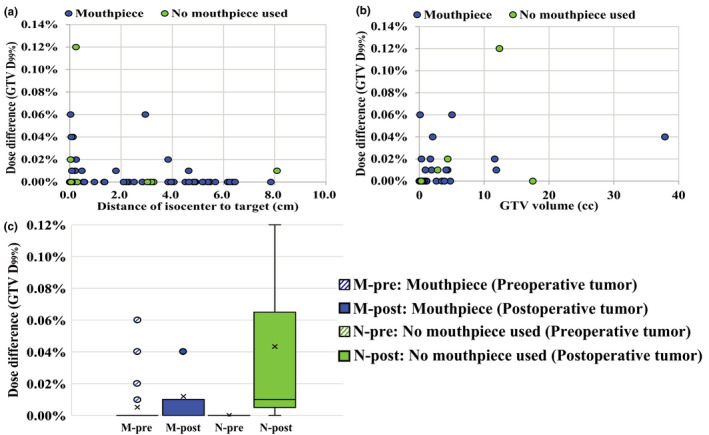
(a) Correlation between dose differences of GTV D_99%_ and distance of isocenter to target. (b) Correlation between dose differences of GTV D_99%_ and GTV volume. (c) Variations in dose differences of preoperative and postoperative tumors. Box plots represent upper and lower quartiles, and the ends of the whiskers represent 1.5× interquartile or maximum ranges. Circles represent outliers, and central solid lines and crosses represent the median and mean values, respectively

Figure 4 shows the results of dosimetry errors due to inter‐ and intrafractional setup errors for GTV D_99%_ and PTV D_95%_ using the SRT and SRS techniques. Dosimetric errors were within 1% for all cases. Figure 5a shows the correlation between the dose differences in GTV D_99%_ and the distance from the isocenter to the target. We observed no clear trends in the distance and dose difference in this study. Figure 5b shows the correlation between the GTV volume and dose differences in GTV D_99%_. The smaller the volume, the larger the dose differences owing to patient setup error. However, dose differences were within 1% regardless of volume when the DSPS and mouthpiece were used.

Figure 5c shows the effect of postoperative and preoperative tumors on dose differences in GTV D_99%_. Although dose differences of the postoperative group were larger than those of the preoperative group, the maximum dose difference remained within 1%. Statistical analysis was not performed because dose differences from planned dose were small in both groups with and without the mouthpiece.

**FIGURE 6:**
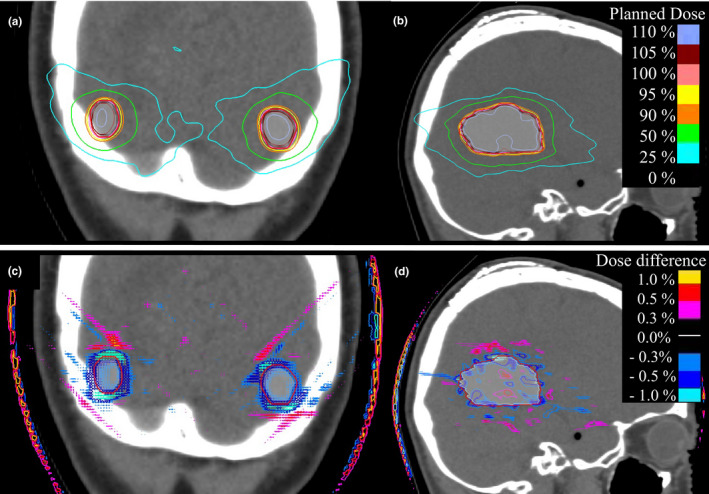
Dose distributions and dose error maps for preoperative (a, c) and postoperative tumors (b, d). White color represents GTV. Red solid line represents the modified PTV. Dose of 30 Gy was normalized to 100% for planned dose, delivered dose, and dose differences. Dose differences were defined as (planned dose–delivered dose)

Figure 6 shows the dose distribution and the dose difference map for patients with preoperative and postoperative tumors. When the preoperative tumors had spherical targets, dose differences occurred at the edge of the target, as seen in Figure 6c. However, when there were postoperative tumors with irregular shapes, the dose distribution within the target was also heterogeneous; therefore, dosimetric errors occurred both inside and outside the target, as seen in Figure 6b,d.

### 3.1 Discussion

Interfractional 3D shift and intrafractional longitudinal setup errors of the mouthpiece group were smaller than those of the no‐mouthpiece group as seen in Tables 2 and 3. This indicates that a thermoplastic mask combined with Precise Bite™ has a higher immobilization accuracy than the case in which only the thermoplastic mask was used. This result consists of previous studies.^6^, ^11^, ^12^ Furthermore, Table 4 indicates that both groups using the DSPS performed higher immobilization accuracy than other immobilization devices.^6^, ^12^ Despite the presence of a significant difference in interfractional 3D error and intrafractional longitudinal setup errors, the difference between the average values of the two groups was 0.1 mm. The resolution of the planning CT was 1 × 1 × 1 mm^3^ and that of the CBCT was 0.5 × 0.5 × 2 mm^3^. A limitation of this study was whether we could statistically analyze a difference in the resolution of the CT images of only 0.1 mm with sufficient accuracy. However, both the DSPS alone and in combination with the mouthpiece have immobilization accuracy that can be used for brain SRS/SRT.

As a result of van Herk's recipe, both groups had PTV margins of <1.5 mm as seen in Table 5. Although the DSPS alone can achieve the same immobilization accuracy as previous studies,^7^, ^11^ the combination of the mouthpiece and the DSPS further improves the immobilization accuracy. Wang et al. investigated the intrafractional PTV margin with the combination of cushion, mask, and mouthpiece.^11^ The calculated PTV margins according to van Herk's recipe were 1.3, 0.9, and 1.3 mm in the lateral, vertical, and longitudinal directions, respectively.^11^ In another study, Naoi et al. evaluated the immobilization accuracy with a mouthpiece‐assisted thermoplastic mask using van Herk's recipe.^7^ Similarly, their study evaluated the PTV margins which were 0.97, 1.30, and 0.88 mm, respectively. Our study showed that the PTV margins calculated with van Herk's recipe were smaller than previous studies. Significant difference was only noted in the longitudinal direction, but the PTV margin calculation yielded improvements in the lateral and the longitudinal directions when the mouthpiece was used. Therefore, the combination of the DSPS and a mouthpiece has a higher immobilization accuracy than the conventional thermoplastic masks. The DSPS is suitable for multitarget SRS in conjunction with a single isocenter technique.

The DSPS is held in place by two thermoplastic boards at anterior and posterior parts of the head. Custom‐made supports at the posterior part of the head for each patient achieved higher immobilization accuracy than the conventional thermoplastic masks. However, the combination of the DSPS and mouthpiece holds the patient very tightly and the patient may feel uncomfortable. One case of a patient whose mouthpiece was painful and the DSPS was recreated in this study. Therefore, radiation oncologists need to carefully assess patient discomfort associated with the use of the mouthpiece.

Previous studies investigated that the rotational positioning error requires an additional PTV margin depending on the distance between the isocenter and targets.^5^, ^13^ The DSPS alone and the combination of the DSPS and the mouthpiece indicated the small rotational errors as shown in Figure 1b and Figure 2b. Then, an additional PTV margin due to the rotation error. In the immobilization devices used in this study, multiple‐targets brain SRS/SRT with the single isocenter does not require additional PTV margin due to the rotational error.

Ohira et al. investigated the plan quality of conventional VMAT and HyperArc VMAT.^20^ They reported that the GI values were 3.06 ± 0.42 and 3.91 ± 0.55 for HyperArc and conventional VMAT, respectively. The GI values of this study were 4.02 ± 0.53 and 3.50 ± 0.13 for PTV volumes greater than 3 cc for the mouthpiece and no‐mouthpiece groups, respectively. Our results were similar to those of previous studies. Similarly, the CI values were reported to be 0.93 ± 0.02 and 0.90 ± 0.05 for HyperArc and conventional VMAT, respectively. The CI values of our study were 0.84 ± 0.11 and 0.81 ± 0.06 for the mouthpiece and no‐mouthpiece groups, respectively. These results indicate that assessment of immobilization accuracy of the DSPS and the mouthpiece was performed using the same steep dose distribution as in the previous study.^20^

Dose differences between planned and delivered doses were within 1%. These results indicate that both the DSPS alone and together with the mouthpiece could ensure the dose coverage of the GTV with a PTV margin of 1 mm. The PTV margin derived in terms of dose delivery was different from the geometrically calculated PTV margin of 1.5 mm for the DSPS alone. Since the dose grid was 1 mm, the impact of inter‐ and intrafractional setup errors on DVH parameters was limited when the shift was within 1 mm/1 degree, as shown in Figure 1. In particular, the dosimetric error of the SRT technique that performs fractionated irradiation was smaller than that of the SRS technique.

This study also evaluated dosimetric errors caused by patient setup errors for irregularly shaped postoperative tumors. Previous studies calculated the PTV margin for multitarget brain SRS/SRT with a single isocenter from a geometric point of view.^13^, ^14^ The limitation of their investigation was that they assumed uniform sphere targets and uniform dose distribution in the calculation of PTV margins. Therefore, investigation of the PTV margin for irregularly shaped postoperative tumors is still required. In this study, we found that both the DSPS and the mouthpiece had good immobilization accuracy with a PTV margin of 1 mm and a dose difference of less than 1% for postoperative tumors.

Mischa et al. investigated that intrafractional setup errors increased linearly with treatment time.^21^ Immobilization accuracy of the DSPS presented in this study can be used for treatment times up to approximately 8 min. When the DSPS is used for longer treatment times, an assessment of immobilization accuracy is required again.

The major limitation of this work was the voxel size. The voxel sizes of planning CT and CBCT were 1.07 mm and 0.51 mm, respectively. Uncertainties existed owing to differences in voxel sizes. Moreover, given that the minimum resolution was 0.5 mm, an error smaller than this value cannot be detected. Although the feasibility of the mouthpiece was indicated for intrafractional setup errors, it is necessary to consider the uncertainty associated with the voxel size.^22^ It ensures that two groups of intrafractional setup errors were smaller than the resolution of CBCT, as seen in Table 3. Although a significant difference is shown in intrafractional longitudinal setup errors, uncertainty existed in the accuracy of data analysis. On the other hand, it has meaning since interfractional setup errors over the resolution of CBCT, as seen in Table 2. In this study, we calculated the PTV margin using a combination of inter‐ and intrafractional setup errors. In addition, we evaluated with delivered dose analysis. Interfractional setup errors affect dosimetric errors more than intrafractional setup errors in these analyses. Therefore, we think that the resolution of imaging limitation is becoming smaller.

Other than systematic and random setup errors, residual errors, such as mechanical errors, and isocenter misalignment should be considered. These were not directly related to the immobilization accuracy of the devices and were thus omitted. However, these errors need to be added as PTV margins in clinical treatments. According to the AAPM TG‐142 report, the mechanical errors performing SRS/SRT are within 1 mm even off‐isocenter target.^23^ To ensure quality assurance of SRS/SRT, deviations should be constantly monitored and adjusted if they can be corrected. The deviation of the facility should be investigated and added to the PTV margin calculation as shown in the PTV margin recipe (II).

## 4 CONCLUSIONS

In terms of geometric assessments, the calculated PTV margins using the DSPS with the mouthpiece were 0.89, 0.75, and 0.90 mm for the lateral, vertical, and longitudinal directions, respectively. Furthermore, the PTV margins in the case the DSPS was used without the mouthpiece were 1.20, 0.72, and 1.37 mm for the lateral, vertical, and longitudinal directions, respectively. In terms of dosimetric assessments, both the DSPS alone and the combination of the DSPS and the mouthpiece were available with a PTV margin of 1 mm in both SRT/SRS techniques. Dose differences within 1% were also investigated for irregularly shaped postoperative tumors. Therefore, the DSPS alone provides stable immobilization accuracy. Even higher accuracy can be expected when the mouthpiece is also used.

## CONFLICT OF INTEREST

No conflict of interest.

## AUTHOR CONTRIBUTIONS

All co‐authors revised the manuscript. Jun Tomihara collected and analyzed the data. Jun Tomihara and Jun Takatsu prepared the manuscript. Jun Takatsu is the corresponding author and designed the study. Satoru Sugimoto and Naoto Shikama provided supervision of the manuscript. Keisuke Sasai provided final approval of the manuscript.
